# From Playroom to Lab: Tough Stretchable Electronics Analyzed with a Tabletop Tensile Tester Made from Toy‐Bricks

**DOI:** 10.1002/advs.201500396

**Published:** 2016-01-13

**Authors:** Richard Moser, Gerald Kettlgruber, Christian M. Siket, Michael Drack, Ingrid M. Graz, Umut Cakmak, Zoltan Major, Martin Kaltenbrunner, Siegfried Bauer

**Affiliations:** ^1^ Soft Matter PhysicsLinz Institute of Technology LITJohannes Kepler University LinzAltenbergerstrasse 694040LinzAustria; ^2^ Institute of Polymer Product EngineeringLinz Institute of Technology LITJohannes Kepler University LinzAltenbergerstrasse 694040LinzAustria

**Keywords:** graded elastomer blend, rapid prototyping, rigid island, stretchable electronics, tensometer, tough bonding

## Abstract

Toy bricks are an ideal platform for the cost‐effective rapid prototyping of a tabletop tensile tester with measurement accuracy on par with expensive, commercially available laboratory equipment. Here, a tester is presented that is not only a versatile demonstration device in mechanics, electronics, and physics education and an eye‐catcher on exhibitions, but also a powerful tool for stretchable electronics research. Following the “open‐source movement” the build‐up of the tester is described and all the details for easy reproduction are disclosed. A a new design of highly conformable all‐elastomer based graded rigid island printed circuit boards is developed. Tough bonded to this elastomer substrate are imperceptible electronic foils bearing conductors and off‐the‐shelf microelectronics, paving the way for next generation smart electronic appliances.

## 1 Introduction

Science presents not only intellectual but also financial challenges.^1^, ^2^ Laboratory equipment in particular is expensive and usually requires large investments—a serious problem in current research with limited financial resources. Building open‐source and customized hard‐ and software is a relatively new trend which enables the development of scientific tools that often meet particular specifications better and at lower cost than commercially available equipment.^3^, ^4^ Many such projects require specialized tools, such as 3D printers, laser cutters, and mills, that may not be readily available to everyone—toy bricks, however, are. LEGO, and the Technic series in particular, is a highly versatile, interlocking construction kit. It combines vast design possibilities with easy and intuitive handling, which makes it the perfect platform for rapid prototyping and educational purposes. Brick‐built constructions are used as sample holders,^5^ as low‐cost replacement for optical tables and components,^6^, ^7^, ^8^ in medical applications^9^ and as rapid‐prototyping equipment.^10^ With the introduction of the Mindstorms series, LEGO extended the system with a programmable brick (LEGO Mindstorms NXT 2.0) that has active sensing and motion capabilities. Its complete integration into the National Instruments' LabView platform (Toolkit available from the NI website^11^) and the availability of open‐source Java plugins^12^ raised the formerly passive system to a multifunctional prototyping and automation system. Realization in LEGO of machinery such as a robot that produces synthetic bone,^13^ a micropump controller^14^ or a high‐precision Watt‐Balance^15^, ^16^ demonstrates its potential for sophisticated tasks.

In this work, we significantly extended the use of toy bricks in the laboratory by developing a uniaxial tensile testing device that is readily employed in highly interdisciplinary research on next generation stretchable electronics. A form of electronics, soft and adoptable, yet sophisticated and functional often requires the able combination of materials with vastly differing properties (i.e., differences in Young's modulus spanning orders of magnitude^17^); a challenge best met with affordable characterization tools. Our tensile tester is perfectly suited to acquire the stress–strain and resistance–strain progressions of stretchable materials and conductors as well as to evaluate the bonding strength between elastomers of tunable stiffness and that of heterogeneous tough bonds of elastomers and polymer foils carrying electronic circuitry. We describe construction, operating principles and limitations of the system and investigated the tunability of the elastic characteristics of silicone elastomer blends (Sylgard 184 and Ecoflex 00–30) in different mixing configurations. Encouraged by the results, we developed all‐elastomer modulus graded rigid‐island structures with a Young's modulus adjustable by a factor of 38 that host a microprocessor‐controlled ultraflexible electronic circuit. Thin metal films (100 nm) on ultrathin polyethylene terephthalate (PET, 1.4 μm) were used as electrical conductors and characterized in terms of long‐term conduction stability at stiff/soft boundaries.^18^ We introduce the strong covalent bonding of such imperceptible electronic foils^19^ with their off‐the‐shelf microelectronics to the engineered elastomer matrix that forms the basis for our multifunctional stretchable electronics platform. Potential applications of our tensile tester are not limited to stretchable electronics research; it is also a perfect demonstration device in mechanics, electronics, and physics education and can be used as an eye‐catcher in exhibitions (Video S1, Supporting Information). For easy reproduction, the schematics of our tensile tester are all open‐source and available from the SoMaP website.^20^

## 2 Results and Discussion

**Figure 1:**
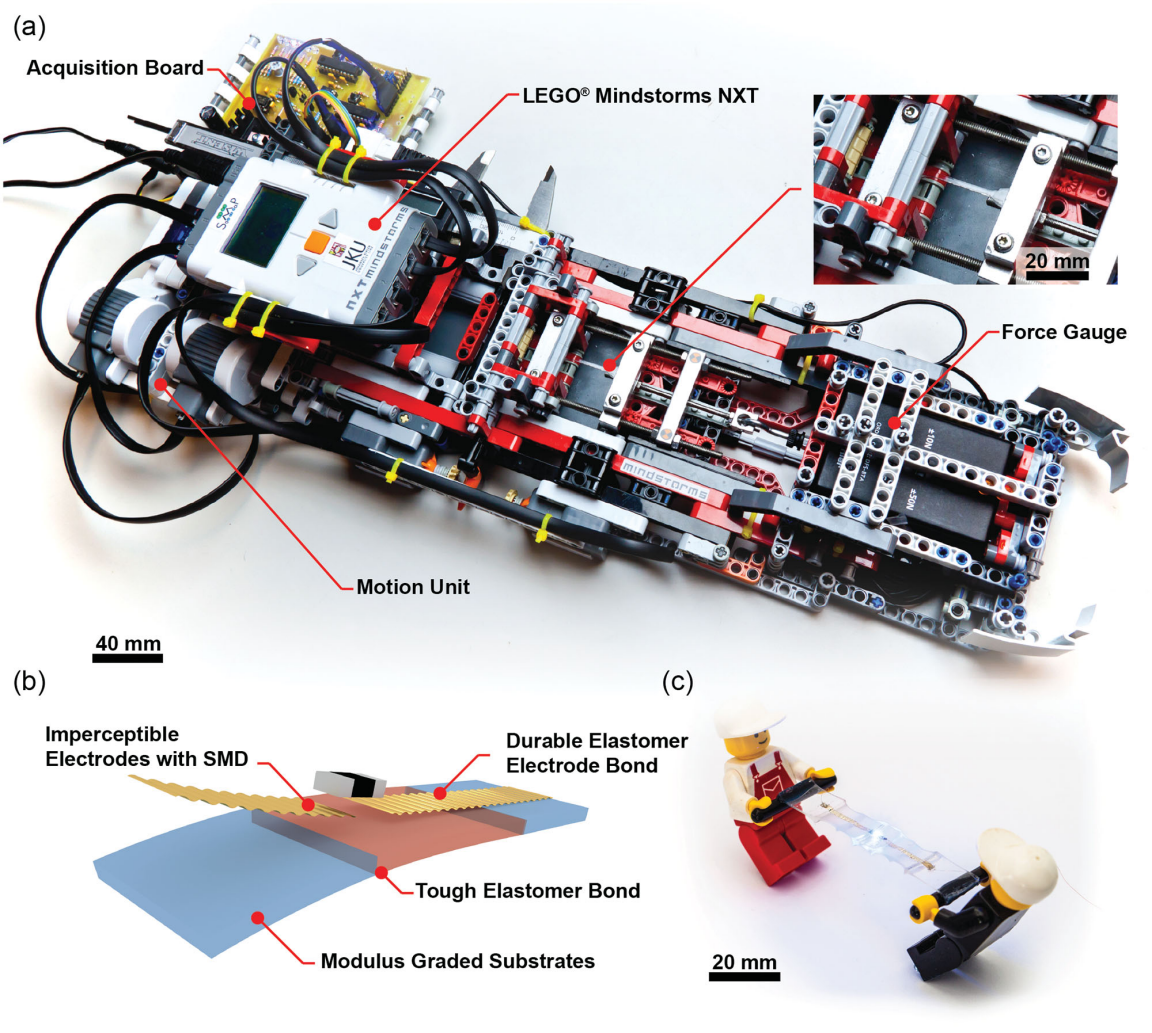
a) Photo of the toy‐brick tensile testing setup. The only elements that are not made from toy bricks are the acquisition board, the clamping device and the digital caliper. The sample is placed in the clamping contraption (see inset for enlarged view) and stretched at constant velocity by the motion unit. b) Illustration of a tough, hybrid stretchable electronic system. PET foils (1.4 μm thick) bearing conductors and surface mounted devices (SMD) are covalently bonded to a prestretched, modulus‐tunable all‐elastomeric substrate with low viscoelasticity. Nonstretchable components (microcontrollers, light emitting diodes (LEDs), etc.) rest at strain‐insulated elastomer islands, the ultrathin PET with metallic interconnects is on deformable regions of the substrate. A reversibly stretchable network of folds and wrinkles forms upon relaxation of the prestretch. c) Hybrid stretchable blue SMD LED kept under tension by two LEGO helpers while operated.

Our toy‐brick setup is simple, portable, and cost‐effective (**Figure** **1**a). It is capable of stretching material samples uniaxially with traveling distances of up to 30 mm while measuring tensile force (≤30 N) and electrical resistance (0–1 MΩ) and concurrently measuring the sample strain. The measurement accuracy of our setup is comparable to that of professional laboratory equipment. It enables us to characterize stretchable conductors by detecting changes in electrical resistance with elongation and to determine their elastic behaviors, which are visualized in resistance–strain and stress–strain plots. The total cost of this instrument amounted to about 700 Euros, which makes it an ideal and cost‐effective tool for the elastic characterization of common elastomers and stretchable conductor designs. The frame of this tabletop machine consists entirely of LEGO Technic parts, providing the necessary mechanical stability and ensuring a lightweight, adaptable, and portable design (more details of the tester are given in Figure S1, Supporting Information). A data acquisition board picks up strain, force, and resistance readings and delivers these data packages on request to the NXT. The NXT then transfers the data via a USB connection to a LabView user interface (National Instruments, USA), where the final data visualization and analysis are performed. Alternatively, an open‐source Java based control and visualization software is possible.^20^ The permanent data flow allows adjustment of all measurement parameters at run time and enables real‐time tracking of the data obtained. These recordings can be gathered either by manual control of the measurement procedures or by executing predefined sequences.

To demonstrate the full potential of our open‐source low‐cost lab equipment, we developed and benchmarked a tough, hybrid stretchable electronic system (Figure 1b). Ultrathin polymer foils (1.4 μm thick) bearing conductors and surface mounted devices (SMD) are covalently bonded to a prestretched, modulus‐tunable all‐elastomeric substrate with low viscoelasticity. This approach allows us to place the nonstretchable components (microcontrollers, light emitting diodes (LEDs), etc.) at stiffer, strain‐insulated elastomer regions, whereas the ultrathin PET foil with metallic interconnects rests on readily deformable regions of the substrate and in turn forms a reversibly stretchable network of folds and wrinkles upon relaxation of the prestretch. An example of this approach is depicted in Figure 1c, where a stretchable blue SMD LED is kept under tension by two LEGO helpers while it is operated.

**Figure 2:**
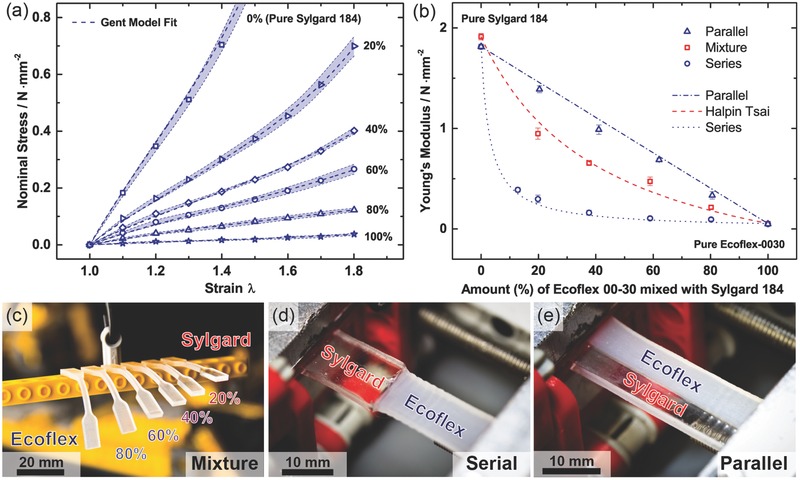
a) Stress–strain behavior of Sylgard 184/Ecoflex 00–30 blend with different mixing ratio. Shown are the arithmetic means of the stress–strain data of four individual samples (blue symbols) of each elastomer blend (20 wt% steps), the ±1σ confidence intervals (blue shaded areas) and the respective Gent fits (blue dashed lines). b) Young's moduli of the blends: experimental values and theoretical modelling using the Halpin‐Tsai composite model, together with experimental and theoretical values for the extreme configurations of purely serial and purely parallel alignment. c) The increasing stiffness of the Sylgard 184/Ecoflex 00–30 blends visualized by gravitational bending. d,e) Combination samples for serial and parallel alignment of the elastomers.

In the following, we describe the materials building blocks, the required strong bonding of these heterogeneous compounds as well as the modeling and electro‐mechanical characterization of this durable stretchable electronic platform. We used the European Standard EN ISO 527‐2 for the elasticity characterization of the engineered silicone elastomer matrices. This norm regulates the testing procedure and sample shape for the determination of tensile properties of plastics.^21^ Throughout our tests we used the shouldered bar specimen type 5B (Figures S2a and S2b, Supporting Information). To obtain the nominal mechanical stress *σ_N_ = F/A_0_* in the uniform region from the tensile force *F*, the initial cross‐section *A_0_* = *d·b* must be known. To this end, the thickness *d* and width *b* of the specimen were determined using a micrometer gauge and a sliding caliper, respectively. We first characterized polydimethylsiloxane (PDMS) with different grades of stiffness resulting from a variation of the prepolymer base to crosslinking agent ratio and benchmark our LEGO tensometer with data obtained using a commercial Bose tensile testing machine (Figures S2c and S2d, Supporting Information). This initial test verifies that our system delivers highly reliable and reproducible results, in excellent agreement with the Bose setup. Given the accuracy and high quality standards of the toy bricks and the use of a calibrated caliper and force gauge, these results are expected and not surprising. In this approach, the degree of interlinking of the elastomer is controlled via the amount of cross‐linker, resulting in different Young's moduli. Changing the base‐to‐cross‐linker ratio is however not a preferable method for tuning the modulus of PDMS, since the stoichiometry of the cross‐linking process is not preserved.^22^ Adding a thinning agent circumvents these issues and allows adjustments of the Young's modulus by about 30%.^23^ Alternatively, the Young's modulus can be tuned over a wide range by mixing two compatible elastomers with different stiffnesses.^24^, ^25^ We fabricated elastomer blends of Sylgard 184 and the much softer Ecoflex 00–30. Depending on the mixing ratio of the blend (Sylgard 184 : Ecoflex 00–30), the Young's modulus is controlled by a factor of 38 from 50 kPa to 1.9 MPa, which practically covers the whole range reported for soft tissues.^17^ Mixing the two silicone elastomers requires no special measures. The raw components of the two elastomers were mixed according to the manufacturers' specifications (10:1 for Sylgard 184 and 1:1 for Ecoflex 00–30) and combined at different ratios (20 wt% steps). The detailed fabrication process is given in the Experimental Section. We fitted the stress–strain data of four samples per mixing ratio (**Figure** **2**a) with the Gent hyperelastic model (Equation (1)) using the Young's Modulus *Y*, the limiting parameter *J*_m_, the rotational invariant *I*_1_ (Equation (2)) and an assumed Poisson‐Ratio of *ν* = 0.5

(1) $$\sigma_{N}=\frac{Y}{3}J_{m}\frac{\lambda -1/\lambda^{2}}{J_{m}-I_{1}+3}.$$

(2) $$I_{1}=\lambda^{2}+\frac{2}{\lambda }.$$

The arithmetic means of the stress–strain data, the ±1σ confidence interval and the Gent fits are plotted in Figure 2a. The high reproducibility of the results demonstrates that this elastomer blend can be engineered with consistent mechanical properties. The resulting Young's moduli (Figure 2b) were fitted to the Halpin‐Tsai elasticity model (Equation (3)),^26^ which was designed for composite materials consisting of a soft filler and stiff reinforcements, using the volume fraction of Ecoflex 00–30 *V_E_*, the reinforcement geometry parameter *ζ*, and Young's moduli for Ecoflex 00–30 *Y_E_* and Sylgard 184 *Y_S_*. The parameter η is a measure of the difference in the Young's moduli of the two components. This enables easy determination of the mixing ratios for Sylgard 184 and Ecoflex 00–30 to create elastomers with arbitrary Young's moduli *Y*_MIX_ within the tunable range. Figure 2c illustrates the large differences in stiffness with samples mounted on a toy brick. We obtained fitting parameters of *Y_S_* = 1.91 MPa, *Y_E_* = 50 kPa, and *ζ* = 0.38

(3) $$Y_{\mathrm{MIX}}=Y_{S}\frac{1+\zeta \eta V_{E}}{1-\eta V_{E}};\;\eta =\frac{Y_{E}/Y_{S}-1}{Y_{E}/Y_{S}+\zeta }$$

Interestingly, when only small amounts of Ecoflex 00–30 (<20 wt%) are mixed into Sylgard 184, a sharp decrease in the Young's modulus from 1.91 MPa to ≈1 MPa is observed, whereas with higher amounts the trend is almost linear. We suppose this may be due to the random microscopic arrangement of the elastomer conglomerate. Thus, a combination of randomly distributed serial and parallel connections between the filler and reinforcement elastomer domains determines the Young's modulus. To visualize the theoretical extremes of purely serial (Figure 2d) and purely parallel alignments (Figure 2e) of the two elastomers, these cases were also tested and the effective Young's moduli (*Y*_PAR_ and *Y*_SER_) of the combinations modeled with a linear stress–strain behavior (Equations (4) and (5), respectively); these are also the limits of Equation (3) for ζ → ∞ and ζ = 0, respectively

(4) $$Y_{\mathrm{PAR}}=Y_{E}\cdot V_{E}+Y_{S}\cdot \left(1-V_{E}\right)$$

(5) $$\frac{1}{Y_{\mathrm{SER}}}=\frac{V_{E}}{Y_{E}}+\frac{\left(1-V_{E}\right)}{Y_{S}}$$

In these cases, the percentage of Ecoflex *V_E_* represents the ratio of its width *b_E_* and length *L_E_* to the whole sample width *b* and length *L* for parallel and serial combination, respectively. The results (Figure 2b) are in excellent agreement with the simple theory. For amounts of the softer Ecoflex 00–30 exceeding 20 wt%, the Young's modulus of the blend is dominated by the parallel connections and decreases almost linearly. For low amounts (<20 wt%) of Ecoflex 00–30, the influence of the serial connections between the components dominates, resulting in a steep increase in the blend's Young's modulus. Again, we benchmarked the experimental results obtained with our LEGO tester to data collected with the Bose system, and found excellent agreement (Figure S3, Supporting Information). Encouraged by their easy handling, perfect mixing and their large difference in Young's modulus, we used a combination of the two elastomers in a novel rigid‐island approach for stretchable electronics.^27^, ^28^, ^29^, ^30^ Although directly stretchable electronic circuits have recently been reported,^31^, ^32^, ^33^, ^34^, ^35^, ^36^ complex systems require off‐the‐shelf electronic components.^37^, ^38^ Since these are rigid and can therefore not be placed in areas that deform to a large extent, they are placed on a carrier (rigid island) that is much stiffer than the rest of the circuit board and interconnected by stretchable conductors sitting on a soft carrier. In most approaches, the rigidity of the islands is achieved by embedding stiff elements such as plastic films, Si carriers or standard PCBs in a stretchable matrix,^18^, ^39^, ^40^, ^41^ or by spatial chemical modification of the substrate elastomer.^42^ In our hybrid approach, microelectronic compounds are affixed on ultrathin PET foils and electrically interconnected by nm‐thick metal conductors deposited directly on the imperceptible foils. This sophisticated flexible electronics platform becomes stretchable when bonded to the prestretched engineered elastomeric substrate in a way where the rigid parts rest on the stiffened elastomer islands, and the interconnects lie on the soft surrounding regions. Upon relaxation of the prestretch, the PET/metal interconnects form an out‐of‐plane wrinkle structure that allows repeated restretching (up to several 100% tensile strain) without strain‐induced changes in electrical resistance. Critical in achieving a durable stretchable electronic platform is a tough interelastomer bonding and a covalent bonding of the heterogeneous electronic polymer foil to the graded elastomer matrix. The first is achieved due to the choice of elastomers with compatible cross‐linking chemistry (here silicone rubbers with platinum‐catalyzed addition curing) as described above. The second, more challenging bonding requires covalent links between PET and the rubber. We achieve this in a two‐step process where we first attach a silane coupling agent (methyltrisiloxanes and orthosilicates) to the oxygen‐plasma activated PET surface and then covalently bond the silanized PET foil to the prestretched silicone elastomer via a silicone‐based glue (mixture of siloxanes and silicones, details on the process see Experimental Section). This new method is advantageous compared to previously reported alternatives where a sticky, often viscoelastic acrylic elastomer serves as dispersive adhesion layer^18^, ^32^, ^35^: It preserves the ease of tunability in elastic properties in a complex structure across the substrate, allows for near‐arbitrary shapes via casting and molding of the prepolymer—crosslinker mixtures, and eases the fixation of the electronic layers as alignment mismatches can be corrected while the elastomer adhesive cures.

**Figure 3:**
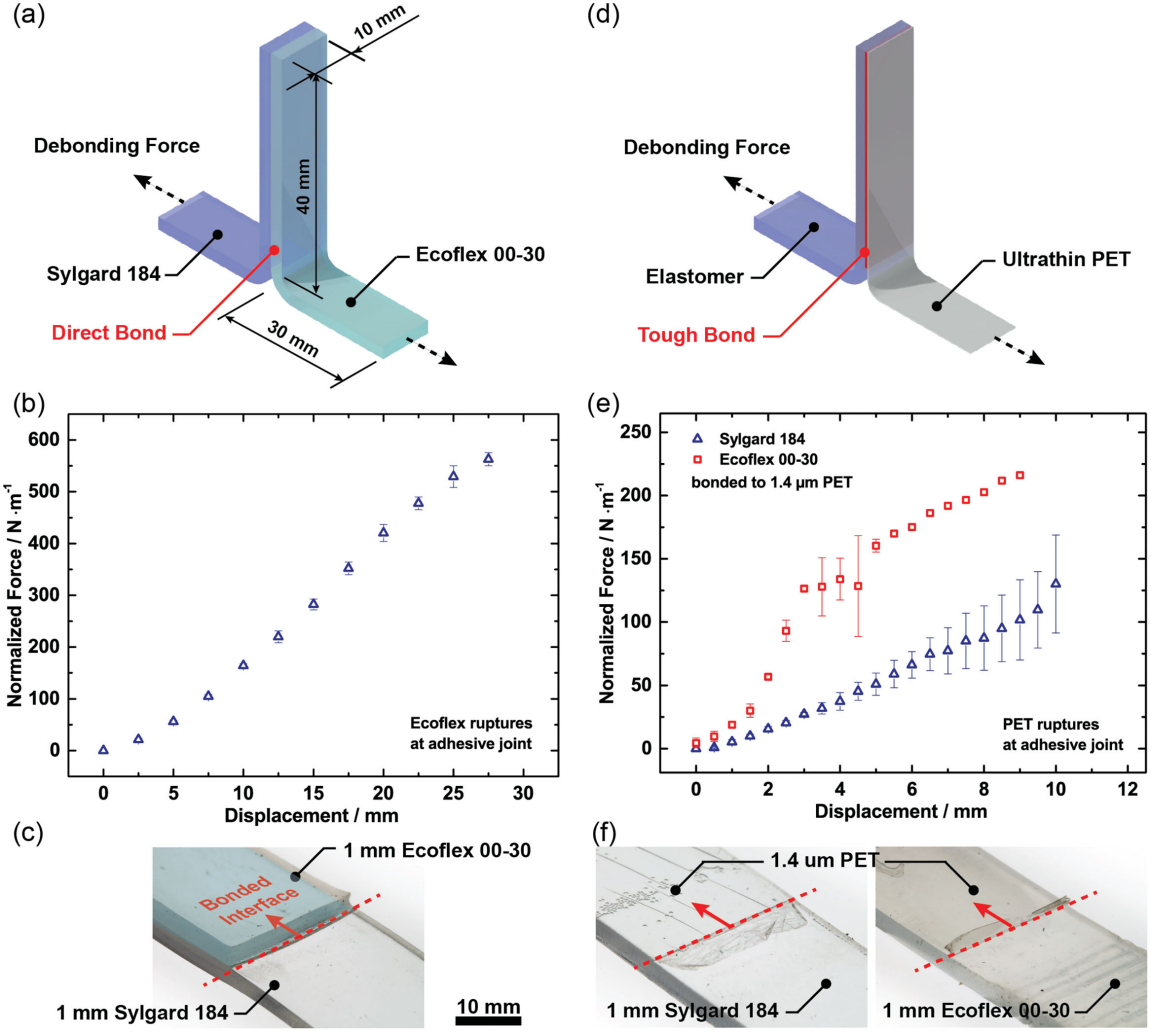
a) Illustration of T‐shaped all‐elastomer specimen for debonding force measurements. Slabs of Sylgard 184 and Ecoflex 00‐30 are directly bonded to form a T, force is applied perpendicular to the bonded area. b) Observed force per sample width (10 mm) versus displacement. Error bars represent standard deviation for five individually measured specimens. The clamped shoulders of the soft elastomer elongate until the rupture strength of the Ecoflex is exceeded (550 ± 33 N⋅m^−1^, lower bound of bonding force). No delamination of the bonded interface occurs. c) Photograph of a specimen showing the failure mode. The Ecoflex 00–30 shoulder ruptures near the bonded interface. d) Illustration of test specimen used to characterize the covalent bonding of 1.4 μm thick PET foil on Sylgard 184 and Ecoflex 00–30. Silanization of the PET surface and a silicone glue enable strong bonding of the heterogeneous materials. e) Normalized force versus displacement for the PET‐Ecoflex 00–30 bond (red open squares), and the PET‐Sylgard 184 bond (blue open triangles). Error bars represent standard deviation for five individually measured specimens. Here, the elastomers initially extend, followed by a region with overlaid plastic deformation of the thin PET foil. In all cases, the PET foil ruptures near the bonded interfaces; no delamination of the bonding was observed. f) Photographs of failure modes for PET‐Sylgard 184 (left) and PET‐Ecoflex 00–30 (right), showing no signs of bond failure.

It is vital to characterize the bonding strength of the interelastomer and tough PET‐elastomer bonds. We prepared T‐peel test structures based on ISO 11339^43^ to evaluate these bonding forces using our LEGO tensile tester. **Figure** **3**a depicts the slightly modified sample geometry for testing the adhesion strength of Sylgard 184 and Ecoflex 00–30 slabs of 10 mm width, 70 mm length, and 1 mm thickness each, directly bonded in a T‐shape across a 400 mm^2^ area. Owing the soft, stretchy nature of the elastomers, we observed an elongation of the clamped, nonbonded ends of the T when applying forces perpendicular to the bonded area (Figure 3b). In this case, the force required to pull the sample apart is given by the Young's moduli of the two elastomers and increases with continued elongation up to the point where either debonding occurs or the intrinsic fracture strength of one of the elastomers is exceeded. We tested five identically prepared specimens and found the Ecoflex 00–30 to rupture in the vicinity of the bonded area (Figure 3c depicts an exemplary sample and the failure mode) in all cases. No indications for debonding were observed. The measured rupture strength of 550 ± 33 N·m^−1^ (rupture force normalized by the width of the adhesive joint) places therefore a lower bound on the bonding force between the elastomers, and compares favorably to reported bonding strengths for PDMS elastomers achievable by other methods.^44^ The heterogeneous covalent bonding of PET foils to either Sylgard 184 or Ecoflex 30–00 was investigated in a similar fashion, with the specimen geometry shown in Figure 3d. Here, applying a force perpendicular to the bonded interface area resulted initially in elongation of the elastomer (Figure 3e), followed by a region with accompanying plastic deformation of the thin PET foil. Ultimately, the plastic foil ruptured near the bonded area where strain concentrates, both for bonds to Sylgard 184 and to Ecoflex 00–30 (Figure 3f shows failure modes of typical specimen). Likewise, in all cases no observable delamination occurred at the bonded areas. Since the fracture strength of PET stripes scales with their thickness, we repeated the debonding tests with identically prepared samples that use 12 μm thick PET foils (Figure S4, Supporting Information). In this case, we observed rupture of the elastomers, but once more no signs of failure of the covalent PET‐elastomer bond. These experiments allowed us to place a lower bound of 380 ± 76 N·m^−1^ on the bonding strength of PET to Sylgard 184 and 467 ± 16 N·m^−1^ on the PET‐Ecoflex 00–30 interface. Relevant for all practical applications, our bonding techniques form interfaces that match in strength the intrinsic fracture toughness of the constituent materials.

**Figure 4:**
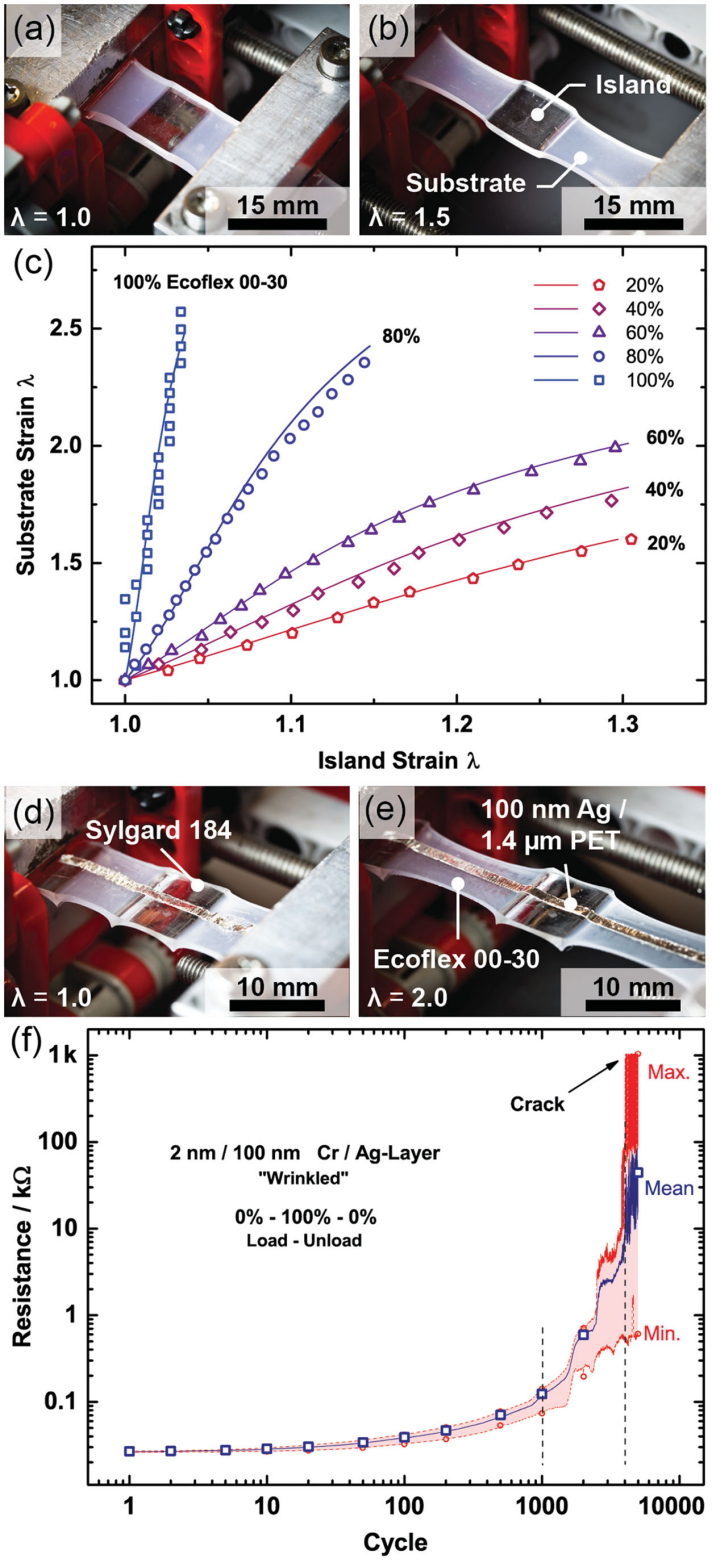
a,b) Rigid 10 × 7 × 2 mm island (Sylgard 184) connected to two 10 × 7 × 2 mm substrate shoulders (Sylgard 184/Ecoflex‐0030 blends) at *λ* = 1 and *λ* = 1.5. c) Strains in island and shoulder, determined by manual measurements (dots) follow the Gent model (lines), which allows the prediction of individual strains in island and shoulders when combining elastomers with different Young's moduli. d‐e) Rigid island configuration with a Sylgard 184 island (10 × 7 × 2 mm) and Ecoflex 00–30 shoulders (10 × 7 × 2 mm) topped with a 1.5 mm wide 2 nm/100 nm Cr/Ag electrode on a 1.4 μm PET foil. The system was continuously stretched to twice its initial length (*λ* = 2) and relaxed at a velocity of 500 μm⋅s^−1^ while monitoring the resistance progression of the electrode. f) Minimum (initial length) and maximum resistance (full elongation) (red) along with the mean over one full load cycle (blue). The initial resistance mean of 26.8 Ω doubled after ≈300 cycles and quadrupled after ≈950 full load cycles. After 4000 cycles, first visible cracks appeared.

Inspired by these findings, we use Sylgard 184 as an island carrier directly embedded in an Ecoflex 00–30 substrate, and a mixture of the two materials as a graded transition between them to realize the engineered elastomeric substrates that host our electronics. The expected elastic behavior of the configuration (**Figure** **4**a,b) is calculated using the Gent model, assuming equal mechanical uniaxial stress in both materials, substrate, and island, and numerically solving for their individual strains under uniaxial deformation (Figure 4c). The findings were verified by stretching with our tensile tester. The individual strains were measured manually with a digital caliper. By introducing a "critical island strain," such as 2%, for the hosted electronic components, the maximum strain of the elastic matrix materials can easily be determined. The gradual strain distribution across the different blends enables smooth transitions in the elastic modulus at the material borders, and minimize electrode or material fatigue.^45^ The stress concentration is dramatically reduced by introducing a gradient layer consisting of 80% Ecoflex 00–30 and 20% Sylgard 184 between island and substrate.

As stretchable electrical interconnect we used bilayers of chromium (Cr) and silver (Ag) with thicknesses of 2 and 100 nm, respectively, on a 1.4 μm thick PET foil.^18^ The fabrication process is described in detail in the Experimental Section. The progression of the resistance with strain, and the endurance and performance over many stretch‐ and relaxation cycles are crucial parameters of such conductors when used at stiff/soft boundaries. We tested the worst‐case scenario by combining a Sylgard 184 island with two Ecoflex 00–30 shoulders topped with a 1.5 mm wide electrode (Figure 4d,e). Since our toy‐brick set‐up is a complete solution for characterizing stretchable conductors, it also allows repeated cycling (stretch–relax) of the specimen while continuously monitoring the electrical resistance (Figure 4f). The initial resistance was measured to be 26.8 Ω. The sample was continuously stretched to full extension (*λ* = 2) at a velocity of *v* = 500 μm·s^−1^ until failure. The electrode resistance was monitored for all cycles, giving information about its progressing degradation. The shadows in Figure 4c indicate the minimum (starting) resistance and the maximum (full elongation) resistance for every cycle, and the dots indicate the arithmetic mean over one cycle. It is remarkable that the system withstands at least 950 full load cycles (4× its initial mean resistance) and remains conductive for up to 4000 cycles, when the first severe cracks appear. The use of a more ductile metallization layer (for example, copper^18^) and neutral mechanical plane configurations^46^ will further improve the cyclic endurance of such stretchable conductors.

**Figure 5:**
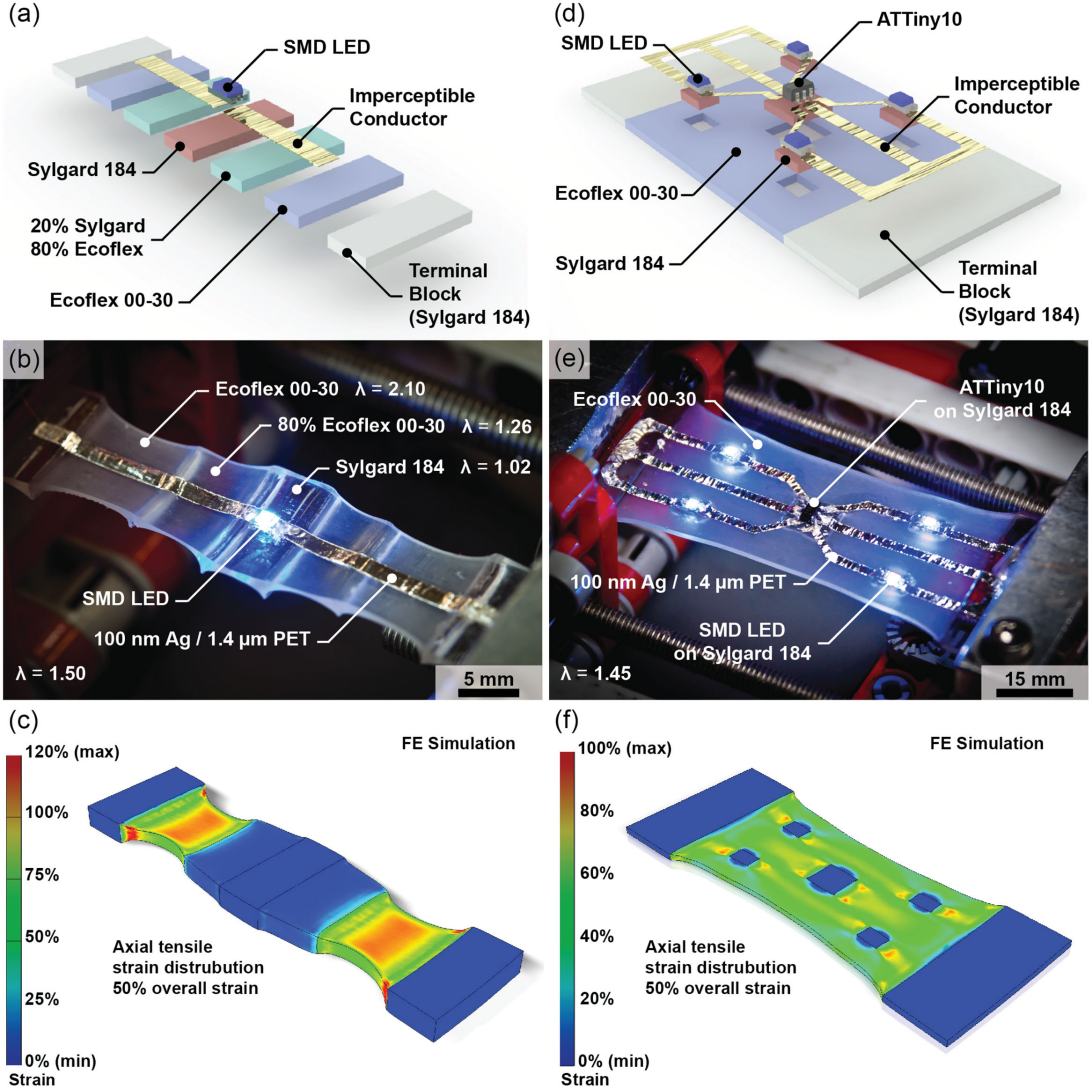
a) Illustration of a stretchable circuit with a surface‐mounted blue LED powered via stretchable Ag interconnects on a modulus‐graded elastomer fabricated with a pure Sylgard 184 island and two shoulders, consisting of 80% Ecoflex 00–30 and pure Ecoflex 00–30. b) Photograph of the elastic circuit while characterized by our tensile tester. The wrinkling of the electrodes guarantees electronic functionality while stretching up to *λ* = 1.5. c) Finite element simulation of the engineered elastomer substrate, illustrating the smooth strain transition between the island and the shoulder layers. d) All‐elastomer circuit board. A surface‐mounted microprocessor (ATTiny10) controls four LEDs. The nonstretchable SMD parts are placed on Sylgard 184 islands embedded in an Ecoflex 00–30 matrix. Imperceptible Cr/Ag form the metallic, stretchable interconnects. e) Photograph of the elastic circuit connected to our toy‐brick setup. The microcontroller drives the LEDs in predefined sequences (Video S1, Supporting Information). f) Strain distribution across the sophisticated elastomeric matrix simulated with a finite element model. Smooth strain transitions from the stiff islands to the soft surroundings prevent electrode failure.

A basic example of a stretchable circuit was realized with a surface‐mounted blue LED powered via stretchable Ag interconnects (**Figure** **5**a). Characterizing this hybrid stretchable system by our tensile tester demonstrates the versatility of our concept (Figure 5b). During operation, a defined current is passed through the circuit. The wrinkling of the electrodes guarantees electronic functionality while stretching up to *λ* = 1.5. The design of the elastomer substrate was guided by finite element simulation in Autodesk Inventor 2014 (Figure 5c), illustrating the smooth strain transition between the island and the shoulder layers. The results encouraged us to fabricate an all‐elastomer circuit board (Figure 5d). Surface‐mounted devices, SMD LEDs and an ATTiny10 (Atmel, USA) microcontroller were placed on Sylgard 184 islands embedded in an Ecoflex 00–30 matrix. Electrical contact throughout the components was achieved via imperceptible Cr/Ag interconnects. By connecting the circuit to our toy‐brick setup the microcontroller awakes and drives the LEDs in predefined sequences, as shown in Figure 5e and Video S1 (Supporting Information).The inbuilt strain limit for this demonstrator was set to *λ* = 1.45 (prestretch on application of the electrodes), while deformation occurred only in the Ecoflex 00–30 regions. The strain distribution across this sophisticated elastomeric matrix was simulated with a finite element model (Figure 5f), showing smooth strain transitions from the stiff islands to the soft surroundings.

## 3 Conclusion

The open‐source tensile tester presented in this work offers an unusual, simple, and cost‐effective approach to scientific measurements by using LEGO Mindstorms and “smart‐sensors” that we designed and built. Our tensile testing setup is capable of uniaxial extension of up to 30 mm at velocities up to 1300 μm·s^−1^. Strain is measured at an accuracy of 100 μm by determining the grip‐grip displacement with a digital sliding caliper. Simultaneous determination of tensile force or resistance and strain enables stress–strain and resistance–strain measurements. For stress–strain studies, a maximum tensile force of 30 N at an accuracy of ±30 mN can be achieved. Resistance–strain measurements can be conducted in a range from 0 to 1 MΩ. We found the performance of our toy‐brick device to be on a par with that of a commercial BOSE tensile tester. Our setup aided the development of a hybrid stretchable electronics platform, where blends of silicone elastomers are engineered to form a complex, structured elastomer substrate with smooth strain transitions between stiff islands in a soft matrix. Covalent bonding of imperceptible electronic foils equipped with off‐the‐shelf microelectronics to such prestretched elastomers results in durable, multifunctional and stretchable electronic circuit boards.

Toy bricks are not only for children. In the hands of engineers, they become a powerful laboratory tool. Since they are almost universally available, toy bricks are an ideal platform for cost‐effective rapid prototyping and related tasks, and for conducting sophisticated and reliable measurements. And a setup that is no longer needed can be torn apart, and something new can be constructed.

## 4 Experimental Section

*Preparation of Sylgard 184, Ecoflex 00–30 and Elastomer Blend Samples Conforming to ISO527‐2/5B for Tensile Testing*: After weighing the Sylgard 184 (Dow Corning, USA) and Ecoflex 00–30 (SmoothOn, USA) components in polypropylene beakers, the raw compounds were mixed for 5 min with a sterile spatula and then degassed in a desiccator to remove air bubbles. The degassed raw elastomer was filled into square‐cut molds (50 × 50 mm, PMMA), followed by another degassing step. The molds were then left to rest for 30 min in a level slot at room temperature to allow the elastomer to form a uniform film. After this leveling process, the mold was cured in the oven for 24 hours at 65 °C. Subsequently, the standardized dumbbell‐shaped (ISO527‐2/5B) samples were cut from the cured films using a laser cutter (Trotec Speedy300). Residues from the cutting process were removed by careful washing with isopropanol and a soft brush. The samples were then dried for 24 h at room temperature. Prior to every tensile test, the fresh samples were stretched and relaxed five times to reduce the influence of the Mullins Effect.

*Preparation of T‐Shaped Samples for Bonding Strength Testing*: For direct bonded T‐shapes of Sylgard 184 and Ecoflex 00–30, the raw components of Sylgard 184 (10:1 wt% base to cross‐linking agent) were weighed in polypropylene beakers, mixed for 5 min with a sterile spatula and then degassed in a desiccator to remove air bubbles. The degassed raw elastomer was filled into a 150 × 70 mm laser‐cut PMMA injection mold with a 1 mm PET separator to form a uniform sheet of elastomer. After precuring the Sylgard 184 for 45 min @ 65 °C, the raw mixed and degassed Ecoflex 00–30 (1:1 for Part A and B, mixing procedure as described above) was injected directly onto the now processable Sylgard 184, enabled by an additional 1 mm PET separator. A lasercut 150 × 30 mm 80 μm polyimide (Kapton, Du Pont) separator coated with SuperSeal (Smooth‐On, USA) was placed onto the Sylgard 184 sheet prior to the injection of the Ecoflex 00–30 to prohibit the bonding of the two elastomers in the region forming the T‐slaps and to create a sharp adhesion border. Curing in an oven for 24 h at 65 °C assured a strong bonding.

Elastomer/PET T‐peel samples use Sylgard 184 and Ecoflex 00–30 prepared as described above. Tough bonding of the PET foils (both 1 μm and 12 μm thickness) to the 1 mm Sylgard 184 and Ecoflex 00–30 elastomer sheets required an oxygen plasma treatment (100 W for 1 min, Glow Research AutoGlow) of the PET surface to form reactive OH‐bonds. Subsequent spin‐coating with a mixture of octamethyltrisiloxane, tetrakis(2‐butoxyethyl) orthosilicate and titanium tetrabutanolate (OS‐1200 primer, Dow‐Corning, USA) at 2000 rpm for 60 s results in a covalent bonding of the PET foil and the silicates. The modified foils are then covalently linked to the elastomer substrates with a thin layer of silicone‐based glue (base and crosslinker system, Pt‐catalyzed addition curing, MED2‐4013, Nusil, USA) applied to the substrates by brush coating. Again a 150 × 30 mm 50 μm Kapton separator coated with SuperSeal is placed prior to the coating with silicone glue. Curing in an oven for 24 h at 65 °C assured strong bonding. After curing, the Kapton separator was removed and 70 × 10 mm samples laser‐cut from the mold. To assure tight clamping of the thin PET‐foils in the LEGO tester, the first 15 mm of the clamping T‐slap were sandwiched between two pieces of VHB4905 (3M, USA).

*Preparation of the Sylgard 184/Ecoflex‐0030 Rigid‐Island Structures*: After weighing the Sylgard 184 (10:1, B:CA) components, the raw compounds were mixed for 5 min with a sterile spatula and then degassed in a desiccator to remove air bubbles. The degassed raw elastomer was filled into square‐cut molds (50 × 50 mm, PMMA) with an additional masking layer to form 7 mm broad elastomer stripes. After another degassing step, the mold was left to rest in a level slot for 30 min at room temperature to allow the elastomer to form a uniform film. Subsequently, the mold was cured in the oven for 45 min. Meanwhile, the raw components of Sylgard 184 and Ecoflex 00–30 were mixed together at the desired weight ratio, stirred for 5 min with a sterile spatula and degassed. After removing the masking layer from the precured but still highly sticky mold, the raw Sylgard 184/Ecoflex 00–30 composite was poured into the blanks of the mold. The mold was then left to rest in a level slot for 30 min at room temperature and cured in the oven for 24 h at 65 °C. The graded structure included one additional step with an 80% Ecoflex 00–30/20% Sylgard 184 blend. After curing, the desired sample geometry was cut from the cured films using a laser cutter (Trotec Speedy300). Residues from the cutting process were removed by careful washing with isopropanol and a soft brush. Subsequently, the samples were dried for 24 h at room temperature.

*Fabrication of the Imperceptible Cr/Ag Electrodes*: The samples were fabricated by thermally evaporating 1.5 × 60 mm chromium‐silver bi‐layer (2 nm/100 nm) stripes on a 1.4 μm PET film through a laser‐cut Kapton shadow mask. Both metals were evaporated at a rate of 0.1 nm·s^−1^. The electrode stripes were laser‐cut, carefully removed from their support, and then bonded to the now prestretched elastomer substrate following the same procedures as described above for the nonelectronic PET foils. Upon relaxation of the samples, the thin electrode film forms wrinkles and stays conductive, since stretching simply causes dewrinkling of the electrode. The resistance remains almost constant until the sample is fully elongated. Electrical contact to the toy‐brick system was achieved by connecting 100 μm thick copper wires to the electrodes with conductive epoxy (Circuit Works, Chemtronics, USA). To guarantee proper conduction, the epoxy was cured for 6 h at 65 °C.

*Fabrication of the Stretchable Circuit Board*: Sylgard 184 rectangles with a thickness of 1 mm and dimensions according to the electronic components (5 × 5 mm for SOT‐23 ATTiny, 3 × 2 mm for 0805 LEDs) to be placed on them were cast in a laser‐cut PMMA mold. After curing for 45 min at 65 °C, the mold was removed and the voids filled with Ecoflex 00–30. The mold was then left to rest in a level slot for 30 min at room temperature and cured in the oven for 24 h at 65 °C. The electrodes were fabricated and affixed to the substrate as described above. The electronic components (microcontroller and SMD LEDs) were fixed to the Sylgard 184 islands by means of a droplet of epoxy adhesive and connected to the electrodes with conductive epoxy (Circuit Works, Chemtronics, USA) under a microscope. Electrical contact to the toy‐brick system was achieved with 100 μm thick copper wires. To guarantee proper conduction, the epoxy was cured for 6 h at 65 °C.

*Measurement Parameters*: To ensure reproducible results, all stress–strain measurements were conducted with a constant travelling velocity of 200 μm·s^−1^, the T‐peel bonding tests with 10 mm⋅min^−1^ and the electrode degradation tests with 500 μm·s^−1^. To avoid slippage we used a clamp tightening torque of 0.3–0.35 Nm on both screws of each clamp for all measurements.

## Supporting information

As a service to our authors and readers, this journal provides supporting information supplied by the authors. Such materials are peer reviewed and may be re‐organized for online delivery, but are not copy‐edited or typeset. Technical support issues arising from supporting information (other than missing files) should be addressed to the authors.

Supplementary

Supplementary
