# Supplementary material for: From Playroom to Lab: Tough Stretchable Electronics Analyzed with a Tabletop Tensile Tester Made from Toy‐Bricks
Source: Adv Sci (Weinh). 2016 Jan 13;3(4):1500396. doi: 10.1002/advs.201500396 (PMC4985695; doi:10.1002/advs.201500396)

Copyright WILEY-VCH Verlag GmbH & Co. KGaA, 69469 Weinheim, Germany, 2016.

ADVANCED SCIENCE
Open Access

Supporting Information

for *Adv. Sci.*, DOI: 10.1002/advs. 201500396

From Playroom to Lab: Tough Stretchable Electronics Analyzed with a Tabletop Tensile Tester Made from Toy-Bricks

Richard Moser, Gerald Kettlgruber, Christian M. Siket, Michael Drack, Ingrid M. Graz, Umut Cakmak, Zoltan Major, Martin Kaltenbrunner, and Siegfried Bauer*

Supporting Information

From playroom to lab: Tough stretchable electronics analyzed with a tabletop tensile tester made from toy-bricks

Richard Moser[1]

Gerald Kettlgruber[1], Christian M. Siket[1], Michael Drack[1], Ingrid M. Graz[1], Umut Cakmak[2], Zoltan Major[2], Martin Kaltenbrunner[1], and Siegfried Bauer[1]

[1] Soft Matter Physics, Johannes Kepler University Linz, Altenbergerstrasse 69, 4040 Linz, Austria.
[2] Institute of Polymer Product Engineering, Johannes Kepler University Linz, Altenbergerstrasse 69, 4040 Linz, Austria.

E-mail: Richard.Moser@jku.at

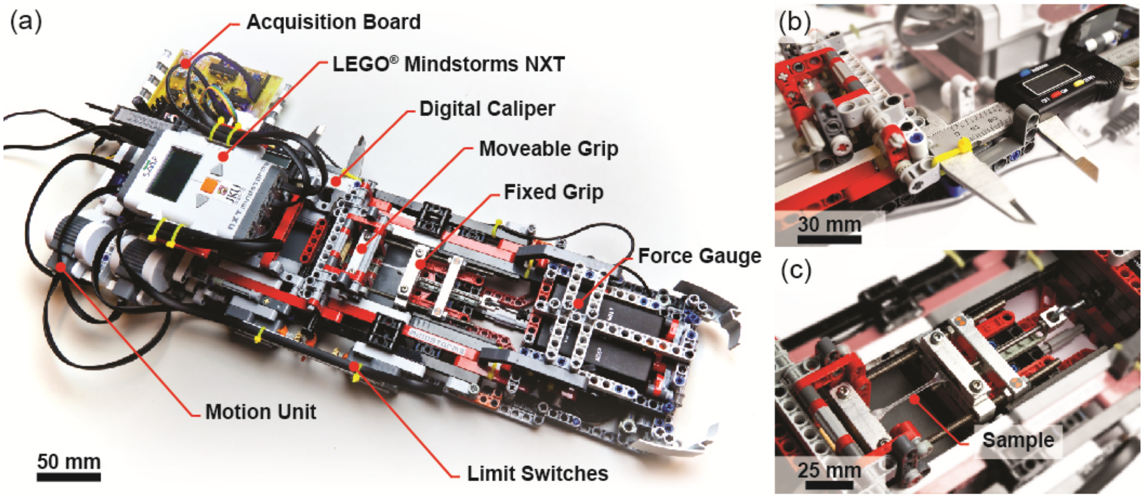

Figure S1 (a) Detailed description of the LEGO® tensile tester. (b) Sample strain is measured with a digital caliper, which tracks the movement of the movable grip. (c) The fixed grip is securely mounted on the force gauge monitoring the tensile force. Electrical resistance is measured directly on the acquisition board, which also picks up the strain and force readings and sends the data to the NXT, which communicates with the LabView control unit.

Uniaxial movement is achieved by a motion stage driven by two coupled and regulated LEGO® NXT Motors and controlled by the programmable LEGO® NXT 2.0 brick. Inbuilt and external gearing increases the torque to achieve constant velocity across the whole load range. Linear (thread-rod) actuators convert the rotary motion of the motors into a linear movement, which ensures a defined velocity ≤1300 $\mu m \cdot s^{-1}$ of the actuated grip. This grip is connected mechanically to a digital sliding caliper. Custom-built readout electronics at the caliper's serial interface enable cost-effective, precise and automated tracking of the clamp displacement. Two limit switches prevent the stage from moving beyond its limits. For measuring the tensile force, the setup contains a force gauge (Vernier Software & Technology, USA) with selectable 10 N and 50 N ranges. The sensor is mounted securely on the LEGO® frame, with its sensor post mechanically connected to the second clamp (SI Figure 1c). Electrical resistance values are acquired by sourcing a constant current through the specimen and reading the voltage drop across it. Six selectable measurement ranges (10 Ω, 100 Ω,..., 1 MΩ) ensure high accuracy across the defined range of 0 – 1 MΩ. The tight clamping of the material samples is a crucial part of the design, as it ensures

reproducible results. Thus, a metal construction with 3D-printed (Makerbot 2X) toothed washers is used to hold the samples in place. Two screws per clamp offer the advantage of a well-defined tightening torque (0.3 – 0.35 Nm), which is essential since elastomers are considered to be incompressible and are therefore squeezed by the clamping. The resulting Poisson stress leads to a significant change in sample length. The final accuracy and measurement ranges of the system together with parasitic effects such as friction, bending moments and frame stability are summarized in Table S1. The system now runs reliably for over a year in our laboratory, without variation in accuracy and without noticeable fatigue. Gluing the LEGO® bricks together is a possibility to even enhance the frame stability. This, in combination with the inherent interconnections between the bricks, will make it at least as tough as a purely 3D-printed or laser-cut frame.

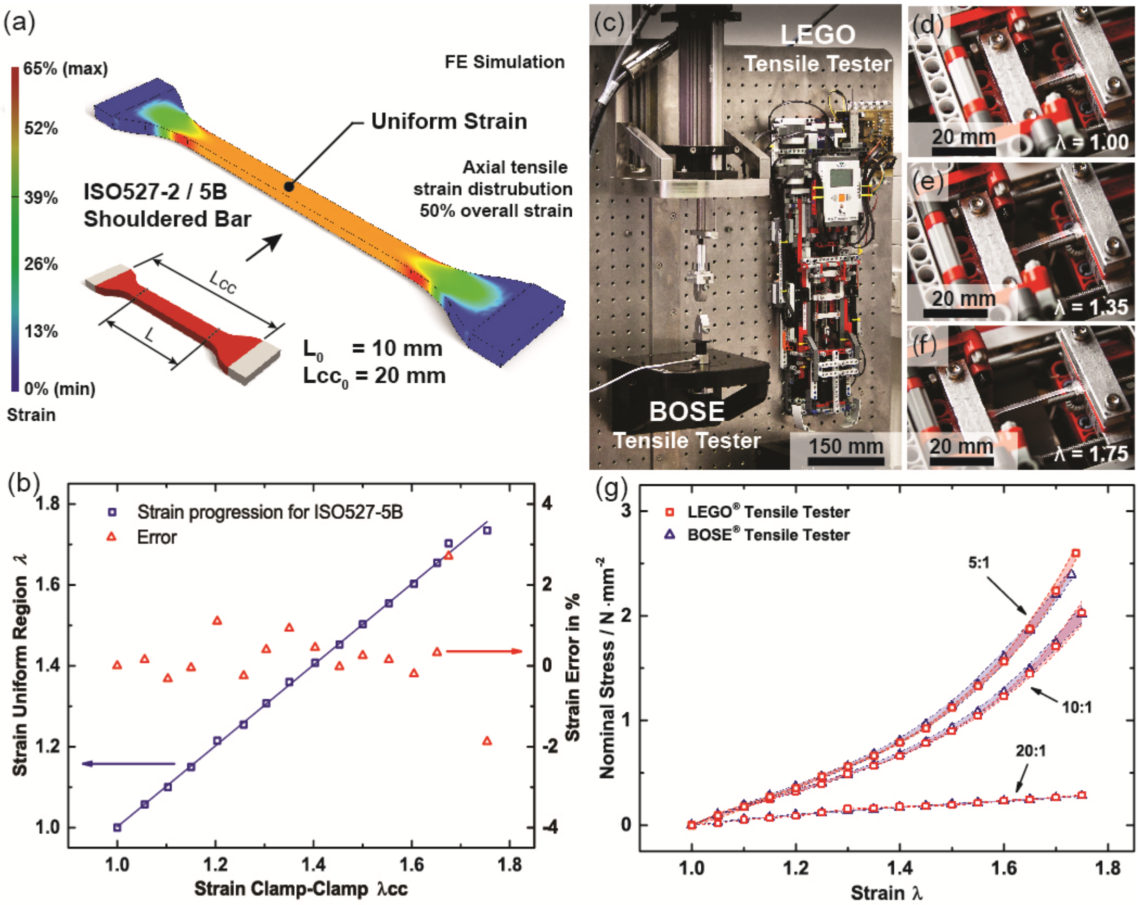

Figure S2 (a) In our stress-strain measurements, we used the ISO527-2 / 5B geometry. It combines a large bearing area with a narrow bar, providing nearly uniform uniaxial straining of the bar. Whereas the strain in the bearing area is highly inhomogeneous, the strain remains constant also for high strains in the narrow bar, which was confirmed by finite-element simulation in Autodesk Inventor 2014. (b) Validation of clamp displacement as a means of determining the sample strain in the narrow bar region. The clamp-clamp displacement ratio λcc directly correlates with the actual strain in the uniform bar λ (blue dots). The blue line indicates the idealized direct correlation curve used ($\lambda = \lambda cc$). Red triangles indicate the deviation (in %) of the strain measurements from the idealized correction. (c) Photo of the toy-brick tensile tester next to a BOSE® test setup. In the tests, the samples were stretched up to λ = 1.75 (d-f). (g) Comparison of the results from the two individual setups based on the stress-strain behavior of Sylgard 184 with different ratios of base (B) to cross-linking agent (CA) (5:1, 10:1, 20:1 – B:CA). The results from the two machines are virtually indistinguishable. The obtained values are also shown in Table S2. In all tensile tests the ISO527-2 / 5B shouldered-bar sample geometry was used.

The ISO527-2 / 5B shape combines a large bearing area with a homogeneous, uniform stress-strain region at the narrow bar at its center. The geometry of this bar is almost unaffected by the sample grips, which squeeze the specimen and therefore create a highly inhomogeneous region in their adjacency. The cross-section remains well defined over the whole uniform region also for high strains, and thus purely uniaxial strain can be assumed in this region. Since the clamp-clamp displacement L_{CC} is needed for calculating the sample strain $\lambda = L_{CC} / L_{CC0}$, where L_{CC0} is the initial clamp displacement, it is important to be aware of the influence of the shouldered bar geometry on the strain progression, because only the uniform bar of the sample provides reliable stress and strain information. Thus, certain correction measures are necessary. By manual measurement we found that the total length L_{CC} is related to the length L of the uniform region by a factor of 0.5 for strains ≤75% (Figure S2b) and therefore $\lambda = \lambda_{CC}$, which was used throughout our measurements. Similar findings were also reported by Schneider et al. for the larger ISO 527-2 / 5A specimen.[23]

Our setup delivers highly reliable and reproducible results. We demonstrate this by comparing stress-strain measurements of polydimethylsiloxane (PDMS) with different grades of stiffness and data obtained using a commercial Bose® tensile testing machine (Figure S2c). The setup was equipped with a long-stroke linear actuator (150 mm travelling distance, 1 kN max. load, Bose Corp., USA), a 22 N load cell (Interface Force Ltd., USA) and WinTest 7 (Bose Corp., USA) analysis software. The elastomer used for the tests, Sylgard 184 (Dow Corning, USA), is a two-component system consisting of a pre-polymer base (B) and a cross-linking agent (CA), and was mixed at weight ratios of 5:1, 10:1 and 20:1 (B:CA). The degree of inter-linking of the elastomer is controlled via the amount of cross-linker, resulting in different Young's moduli. A higher proportion of cross-linker results in a stiffer elastomer. Three samples of each stiffness grade were stretched to $\lambda = 1.75$ (Figure S2d-g) at a constant velocity of 200 $\mu m \cdot s^{-1}$.

As expected, a lower amount of cross-linking agent causes a significant decrease in the Young's modulus, since the change in crosslink density results in a less densely meshed network and therefore a softer material. Remarkably, the values acquired

using the toy-brick setup are in excellent agreement with those from the professional machine (Supplementary Video S1): Not only the initial trend, but also the whole progress of the stress-strain behavior is identical. Furthermore, the Young's modulus of 10:1 Sylgard 184 matches the findings of Palchesko et al. and Schneider et al.[22,23] However, changing the base-to-cross-linker ratio is not a suitable method for tuning the modulus of PDMS, since the stoichiometry of the cross-linking process is not preserved.[22]

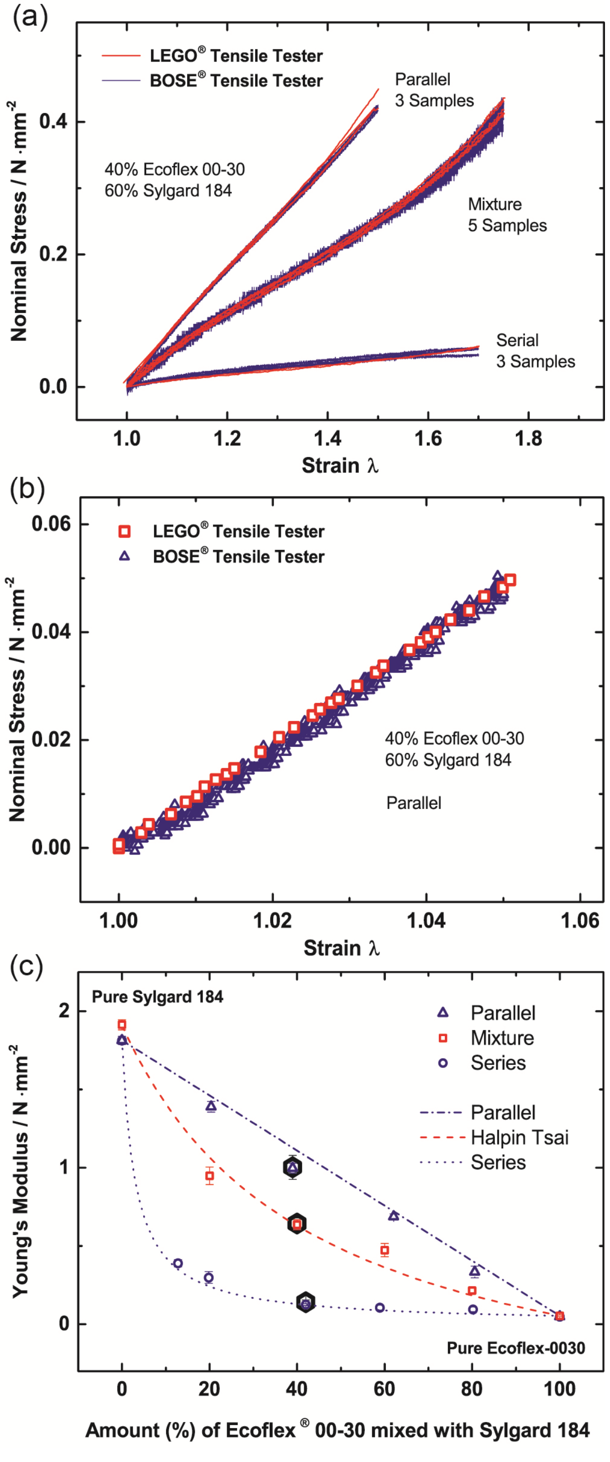

Figure S3 (a) Stress-strain curves recorded for 40% Ecoflex 00-30 and 60% Sylgard 184 elastomer compositions in the parallel arrangement (3 samples each), as a mixture (5 samples each) and in the serial arrangement (3 samples each) using our LEGO® system and the BOSE® tensile tester as benchmark. The measurements are in excellent agreement, demonstrating the reliability and precision of our table top

toy-brick tester. (b) Stress-strain curve of such a sample in parallel arrangement in the small strain regime, with exceptional agreement between the LEGO® and BOSE® systems. (c) Comparison of the Young's modulus obtained with the BOSE® system (black empty hexagons, data from Figure S3a) and data obtained with the LEGO® stretcher (data from Figure 2a and S3a)

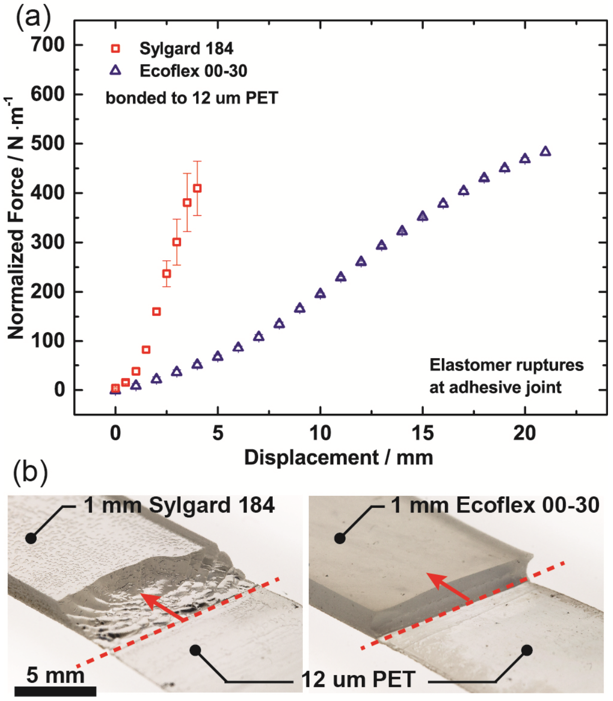

Figure S4 (a) Evaluation of bonding strength for 12-µm-PET – Sylgard 184 and 12-µm-PET – Ecoflex 00-30 tough bonds with T-shaped specimen. Error bars represent standard deviation of the force-displacement behavior over 5 individually tested samples for each bonding type. Applying a force perpendicular to the bonded interface area resulted in elongation of the elastomer until the fracture strength of the rubbers is exceeded. Here, Sylgard 184 fails in an erratic way with multiple fracture sites, as depicted in (b), left photograph. Ecoflex 00-30 ruptures at a single site, close to the bonded area (right photograph in (b). In all cases, the bonded interface showed no signs of fatigue. The bonding forces are thus larger than the fracture strength of the elastomers (380 ± 76 N·m^{-1} for Sylgard 184 and 467 ± 16 N·m^{-1} for Ecoflex 00-30.

Table S1 Performance specifications of the LEGO® tensile tester

Quantity	Range	Resolution
Clamp displacement	L_0 + 30 mm	100 µm
Force	0 - 10 N	30 mN
	0 - 30 N	150 mN
Resistance	0 - 1 MΩ	0.1% of selected range

Table S2 Comparison of Young's moduli in N·mm^{-2} for different stiffness grades of Sylgard 184 by variation of the base to crosslinker ratio.

Mixing ratio (B:CA)	5:1	10:1	20:1
LEGO® setup	2.23 ± 0.04	1.90 ± 0.05	0.56 ± 0.01
BOSE® setup	2.26 ± 0.06	1.89 ± 0.06	0.56 ± 0.02

Supplementary Video S1

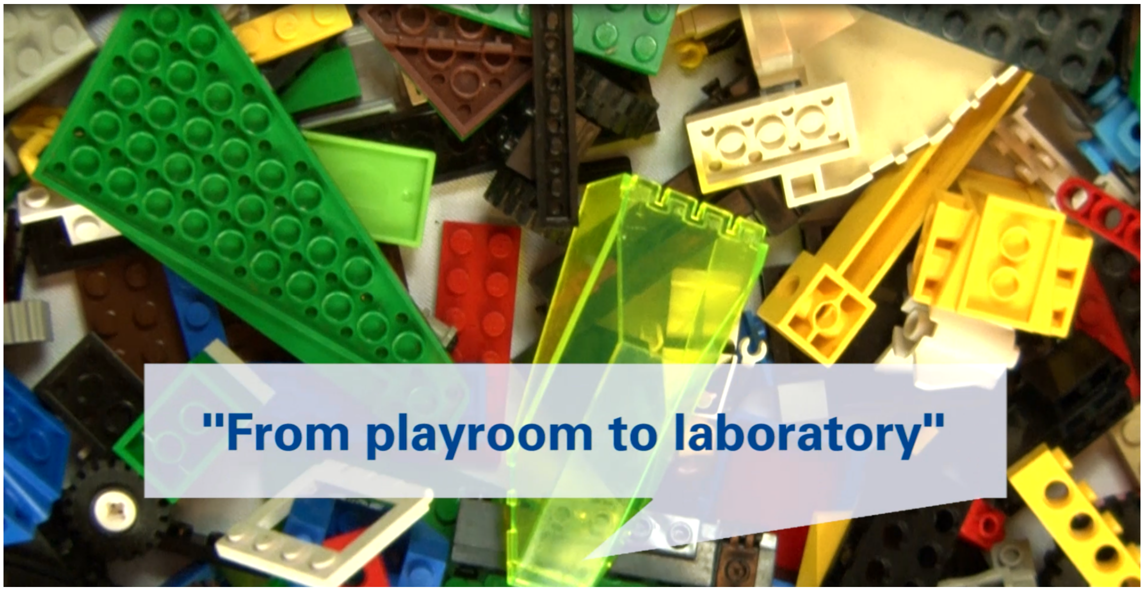

Supplement: Supplementary file 1 — Supplementary [file ADVS-3-0g-s001.pdf]
